# Clinical utility and diagnostic value of tumor-educated platelets in lung cancer: a systematic review and meta-analysis

**DOI:** 10.3389/fonc.2023.1201713

**Published:** 2023-07-26

**Authors:** Elvan Wiyarta, Darrin Ananda Nugraha, Muhammad Indera Ramadani, Gita Fajri Gustya, Muhammad Farrasy Ammar, Hana Dzakira Edwar, Nildza Kheirizzad, Mutiah Nurul Mukhlisah, Erlina Burhan, Elisna Syahruddin

**Affiliations:** ^1^ Respiratory and Tuberculosis Research and Training Center (SATURATE), Faculty of Medicine, Persahabatan National Hospital, Universitas Indonesia, Jakarta, Indonesia; ^2^ Infection Division, Department of Pulmonology, Faculty of Medicine, Universitas Indonesia, Persahabatan National Hospital, Jakarta, Indonesia; ^3^ Oncology Division, Department of Pulmonology, Faculty of Medicine, Universitas Indonesia, Persahabatan National Hospital, Jakarta, Indonesia

**Keywords:** diagnosis, review, liquid biopsy, RNA, biomarker

## Abstract

**Background:**

The review addresses the knowledge gap concerning the diagnostic value and clinical utility of tumor-educated platelets (TEPs) in adult patients with lung cancer.

**Methods:**

We searched twelve databases: PubMed, CENTRAL, EMBASE, CINAHL, MEDLINE, Scopus, ProQuest, MedRxiv, BioRxiv, SSRN, Clinicaltrials.gov, and CNKI up to 24 March 2023, to include any diagnostic study regarding TEPs and LC. TEPs diagnostic value was evaluated from pooled sensitivity and specificity, positive likelihood ratio (PLR), negative likelihood ratio (NLR), diagnostic odds ratio (DOR), and the area under the curve (AUC). QUADAS 2 was used to assess the risk of bias. Heterogeneity analysis was assessed using the receiver operating characteristic (ROC) plane, Galbraith plot, bivariate boxplot, sensitivity analysis, and meta-regression. TEPs clinical utility was evaluated from Fagan’s nomogram.

**Results:**

44 reports from 10 studies, including 7,858 events and 6,632 controls, were analyzed. The pooled sensitivity, specificity, PLR, NLR, and DOR were 0.80 (95% CI 0.79–0.80), 0.69 (95% CI 0.69–0.70), 2.92 (95% CI 2.50–3.41), 0.26 (95% CI 0.21–0.32), and 12.1 (95% CI 8.61–16.76), respectively. In addition, the AUC of the Summary ROC curve was 0.85 (95% CI: 0.81-0.88). The overall risk of bias was low. Heterogeneity may result from cancer stage, cancer control, measuring equipment, and RNA types across studies. There was no apparent publication bias (p=0.29) with significant positive (79%) and negative (22%) post-test probability, according to Deeks funnel plot asymmetry test and Fagan’s nomogram.

**Conclusion:**

TEPs could be a moderately effective candidate biomarker for LC diagnosis.

## Introduction

Lung cancer (LC) remains the leading cause of cancer-related deaths worldwide, representing approximately 18.4% of worldwide cancer deaths (1). LC is classified into two main types: non-small cell lung cancer (NSCLC) and small cell lung cancer (SCLC) (2). NSCLC accounts for about 83% of LC cases, while SCLC accounts for approximately 13% (2). LC must be diagnosed early because it is an invasive and rapidly progressive disease (3, 4). Open surgical tissue biopsy is the current gold standard for diagnosing LC, but this procedure is invasive and associated with high failure rates for mutation evaluation (3, 5). Furthermore, obtaining a tissue biopsy from a single tissue location offers a restricted view of the disease, which may not capture the complexity and diversity of the Stumor (6–8).

Liquid biopsy has emerged as a valuable method for diagnosing LC because it offers the advantages of easier accessibility and more excellent coverage of cancer heterogeneity (8, 9). Blood samples for liquid biopsy can be analyzed for circulating tumor cells (CTCs), cell-free DNA (cfDNA), circulating tumor DNA (ctDNA), and tumor-educated platelets (TEPs) (10). Much research has focused on CTCs, ctDNA, and cfDNA, but these components have limitations. CTCs are challenging to detect due to their rarity and the need for an adequate number of tumor cells and multiple blood samples (11). ctDNA is less stable and has a short half-life (12). Lastly, quantitative cfDNA analysis has unsatisfactory sensitivity (13).

TEPs are a form of liquid biopsy that is becoming an increasingly popular research topic. TEPs are a type of platelet that has been exposed to the cancer microenvironment and subsequently modified to promote cancer growth and progression (14–16). When tumor cells proliferate, they release signals that activate platelets, causing them to undergo morphological and functional changes (14–16). Cancer sequestering extracellular vesicle (EV)-derived ribonucleic acid (RNA) and which alters the platelet RNA profile (17). Directly or indirectly, cancer cells can affect the RNA content of platelets, resulting in the “education” of platelets mediated by the tumor (14). As a novel source of potential biomarkers, alterations in the platelet RNA profile induced by tumors have been described (14). Individual platelet RNA biomarkers and complex RNA signatures can be used for early cancer detection and treatment monitoring (14, 18).

However, numerous TEPs studies, particularly those on LC, remain equivocal and inconclusive (19). Diagnostic studies involving TEPs with LC are difficult to conclude due to the diversity of methods and types of RNA employed, the small sample size, and the limitations of the research design. A comprehensive analysis of this beneficial biomarker’s diagnostic value and clinical utility is crucial. This first comprehensive systematic review and meta-analysis on the diagnostic value and clinical utility of TEPs in LC was conducted to address this knowledge gap.

## Methods

### Search strategy

This systematic review and meta-analysis followed the Preferred Reporting Items for Systematic Reviews and Meta-analyses guidelines (PRISMA) (20). We systematically searched the literature in twelve databases: PubMed, Cochrane Controlled Register of Trials (CENTRAL), Ovid EMBASE, EBSCO Cumulated Index to Nursing and Allied Health Literature (CINAHL), Ovid MEDLINE, Scopus, ProQuest, MedRxiv, BioRxiv, Social Science Research Network (SSRN), Clinicaltrials.gov, and China National Knowledge Infrastructure (CNKI) up to 24 March 2023. The literature on TEPs for the diagnosis of LC was retrieved using the following general search strategies: (tumor-educated platelet) AND (LC) AND (diagnosis OR specificity OR sensitivity OR receiver operating characteristics OR ROC). The general searching strategy is used by first adjusting it to the format in each database, which can be seen in **Supplementary Material S1**, along with the Participant, Index test, Comparison, and Outcome (PICO) for this study (**Supplementary Material S2** **).** We also searched the reference lists of relevant research, systematic reviews, and meta-analyses to identify missed articles during the initial search. Two independent reviewers (DN and MR) independently examined all identified studies. All disagreements are resolved through discussion with the third reviewer (EW).

### Inclusion and exclusion criteria

Inclusion criteria were: (1) diagnostic study design, (2) studies that include TEPs assessment for LC, (3) human-based studies, and (4) absolute numbers of true-positive, false-positive, or false-negative, or true-negative could be calculated from the study. On the other hand, the exclusion criteria were: (1) non-human subject research, (2) case report/series, (3) commentary/viewpoint, (4) narrative review, and (5) irretrievable full-text article.

### Data extraction

Two authors (D.A.N. and M.I.R.) independently extracted data from the full texts of all included studies. The following data were extracted: first author, year of publication, publication location, ethnicity, sample size, age, gender, cancer type, cancer stage, laboratory methods, primer, gene symbol, RNA type, sensitivity, specificity, true positive, false positive, false negative, and true positive.

### Quality assessment

Two independent evaluators (D.A.N. and M.I.R.) assessed the quality of these studies using the Quality Assessment of Diagnostic Accuracy Studies-2 (QUADAS-2) framework, which consists of four domains: patient selection, index test, reference standard, and flow and timing. Each field is used to assess bias, and the first three domains were used to evaluate the applicability of these studies to clinical practice. The likelihood of prejudice and mistrust was categorized as “low,” “high,” or “unclear.” All researchers engaged in discussions to resolve the differences.

### Statistical analysis

Meta-analyses were performed using Review Manager 5.3 (Cochrane Collaboration, Oxford, England), STATA 12.0 (Stata Corp LP, TX, USA), and Meta-DiSc 1.4 (Romany Cajal Hospital, Madrid, Spain). The sensitivity, specificity, positive likelihood ratio (PLR), negative likelihood ratio (NLR), diagnostic odds ratio (DOR), and corresponding 95% confidence intervals (CI) were used to ascertain the diagnostic value of TEPs in LC in a meta-analysis of diagnostic accuracy. The area under the curve (AUC) of the summary receiver operating characteristic curve (SROC) was determined to quantify the diagnostic accuracy. The SROC curve method is a meta-analysis of multiple distinct detection index investigations. By fitting the SROC curve, diagnostic accuracy is comprehensively evaluated based on the weight of their odds ratio.

The analyses for heterogeneity were conducted using the Q test and I2 statistics. P values less than 0.05 were considered statistically significant. A random effects model was employed when I2 > 50% and p-value <0.05 indicated considerable heterogeneity between the included studies. Otherwise, if there was no apparent heterogeneity, the fixed effects model was applied to evaluate the aggregated results. The heterogeneity caused by the threshold effect was assessed using the ROC plane. Galbraith plot and bivariate boxplots were utilized to determine the heterogeneity level. Subgroup analysis and meta-regression were used to evaluate the source of heterogeneity. Individual subgroup results were examined.

Using sensitivity analysis, the reliability of the results was determined. Our results were validated using a variety of statistical tests, including goodness-of-fit, bivariate normality, influence analysis, and outlier detection. The clinical value of TEPs as a diagnostic method was determined using Fagan’s nomogram. Deeks funnel plot asymmetry test was used to examine potential publication bias. A p-value greater than 0.1 indicates the absence of publication bias.

## Results

### Literature searching and study screening

**Figure 1:**
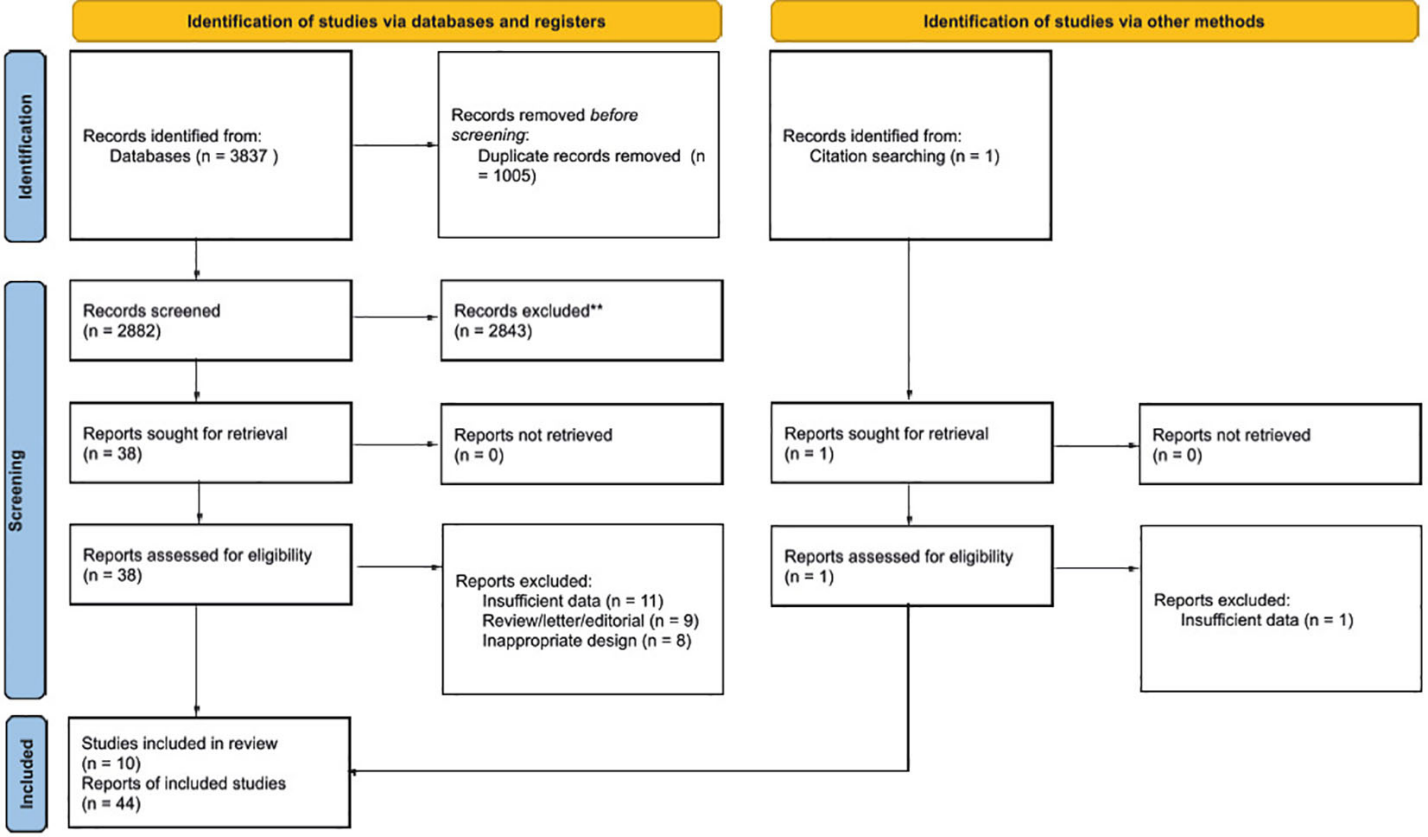
Preferred Reporting Items for Systematic Reviews and Meta-Analyses (PRISMA) 2020 flow chart of the study selection process.

We searched 3837 articles in total from PubMed(723), CENTRAL(148), EMBASE(831), CINAHL(222), MEDLINE(353), Scopus(954), ProQuest(259), MedRxiv(98), BioRxiv(218)), SSRN(4), Clinicaltrials.gov(7), and CNKI(20). Of these, 3848 records were excluded after reading the title and abstract due to the following: duplication, not describing TEPs with LC, not a human model, or studies based on databases. Subsequently, 38 articles remained. After reading the entire text, 28 articles were excluded because of insufficient data, review/letter/editorial, and inappropriate design. Apart from going through the database, a literature search was also carried out through a citation search for the included papers. Citation search retrieves and assesses a study that is then included. Thus, ten studies were included (21–30). Of these ten studies, some had presented more than one report, so the total number of reports included in this review was 44. A flowchart of the whole selection process is shown in **Figure 1**.

### Study characteristics and quality assessment

This review analyzed 14,490 patients, 7,858 events, and 6,632 controls. Of all the studies included, most were conducted in an Asian population (22, 24–30), dominated by men aged around 40 and involving various LC stages. All information regarding the demographic and clinical characteristics of the included studies can be seen in **Supplementary Material S3**. This review also summarizes the laboratory methods and primers used in each study, including platelet isolation, RNA quality assessment, RNA detection, RNA extraction, reverse transcription, PCR, and primers. These results can be seen in **Supplementary Material S4, S5**. The quality assessment is shown in **Supplementary Material S6**. In this review, the domain of patient selection, index test, and flow and timing has a high risk of bias. On the other hand, the domain of patient selection, index tests, and reference standards has a low applicability concern. The outcome summary of the included studies in the meta-analysis is shown in **Table 1**.

**Table 1:** Outcome Summary of Studies Included in Meta-Analysis.

Author, year	Ethnicity	Gene Symbol	RNA Type	Event	Control	Total	Cancer Type	Control Type	Sensitivity	Specificity
**Best, 2017 (** 21 **)**	Caucasian	thromboSeq	vary	85	33	118	Early NSCLC	HS	72.10%	88.90%
				381	76	457	Late NSCLC	HS	88.10%	84.90%
**Luo, 2018 (** 22 **)**	Chinese	MAGI2-AS3	lncRNA	68	60	128	AD	HS	77.90%	81.70%
				33	60	93	SCC	HS	78.70%	88.30%
		ZFAS1	lncRNA	68	60	128	AD	HS	75.00%	66.70%
				33	60	93	SCC	HS	93.90%	43.30%
**Sheng, 2018 (** 23 **)**	Caucasian	48-genes biomarker panel	vary	402	231	633	NSCLC	HS	92.50%	82.70%
**Xue, 2018 (** 24 **)**	Chinese	ACIN1	mRNA	156	58	214	LC	HS	82.70%	44.80%
**Liu, 2019 (** 25 **)**	Chinese	MAX+MTURN+HLA-B	mRNA	127	82	209	LC	HS	60.6%	81.7%
				33	82	115	Early LC	HS	72.70%	85.40%
**Xing, 2019 (** 26 **)**	Chinese	ITGA2B	mRNA	131	45	176	NSCLC	HS+BPN	91.20%	56.50%
				102	21	123	NSCLC	BPN	91.20%	59.40%
				84	42	126	NSCLC stage I	HS+BPN	87.80%	56.50%
				49	24	73	NSCLC stage I	BPN	87.80%	59.40%
		SELP	mRNA	125	51	176	NSCLC	HS+BPN	96.70%	43.10%
				102	21	123	NSCLC	BPN	96.70%	43.80%
				75	51	126	NSCLC stage I	HS+BPN	93.10%	43.10%
				52	21	73	NSCLC stage I	BPN	93.10%	43.80%
**Dong, 2020 (** 27 **)**	Chinese	RNU1	snRNA	382	361	743	NSCLC	HS	74.60%	66.50%
				80	361	441	Early NSCLC	HS	90.00%	38.50%
		RNU2	snRNA	382	361	743	NSCLC	HS	81.40%	74.20%
				80	360	440	Early NSCLC	HS	78.80%	74.20%
		RNU5	snRNA	534	209	743	NSCLC	HS	90.10%	63.70%
				80	361	441	Early NSCLC	HS	86.30%	60.70%
		Combination	snRNA	382	361	743	NSCLC	HS	85.90%	70.10%
				80	361	441	Early NSCLC	HS	93.80%	60.70%
**Yao, 2020 (** 28 **)**	Chinese	CD274	circRNA	562	6	568	NSCLC	HS	90.91%	83.91%
		ITGA2B	circRNA	561	7	568	NSCLC	HS	98.93%	79.31%
		TIMP1	circRNA	509	59	568	NSCLC	HS	89.61%	78.16%
		FLNA	circRNA	516	7	523	NSCLC	HS	90.91%	83.91%
**Dong, 2021 (** 29 **)**	Chinese	SNORD55	snoRNA	290	189	479	NSCLC	HS	79.30%	68.30%
				91	189	280	Early NSCLC	HS	91.20%	49.70%
				204	189	393	AD	HS	77.90%	68.30%
				68	189	257	Early AD	HS	89.70%	49.70%
				76	189	265	SCC	HS	72.40%	77.70%
				19	189	208	Early SCC	HS	68.40%	93.10%
**Li, 2021 (** 30 **)**	Chinese	linc-GTF2H2-1	lncRNA	167	202	369	LC	HS	68.30%	81.70%
				47	202	249	Early LC	HS	69.60%	81.70%
		RP3-466P17.2	lncRNA	167	192	359	LC	HS	77.80%	67.80%
				47	202	249	Early LC	HS	87.20%	55.90%
		lnc-ST8SIA4-12	lncRNA	167	202	369	LC	HS	73.70%	69.80%
				47	202	249	Early LC	HS	83.00%	68.30%
		Combination	lncRNA	167	202	369	LC	HS	82.60%	87.10%
				47	202	249	Early LC	HS	93.60%	69.80%

AD, adenocarcinoma; BPN, benign pulmonary nodules; circRNA, circular ribonucleic acid; HS, healthy subject; LC, lung cancer; lncRNA, long non-coding ribonucleic acid; mRNA, messenger ribonucleic acid; NR, not reported; NSCLC, non-small cell lung cancer; RNA, ribonucleic acid; SCC, squamous cell carcinoma; snoRNA, small nucleolar ribonucleic acid; snRNA, small nuclear ribonucleic acid.

### Diagnosis value of TEPs for LC

**Figure 2:**
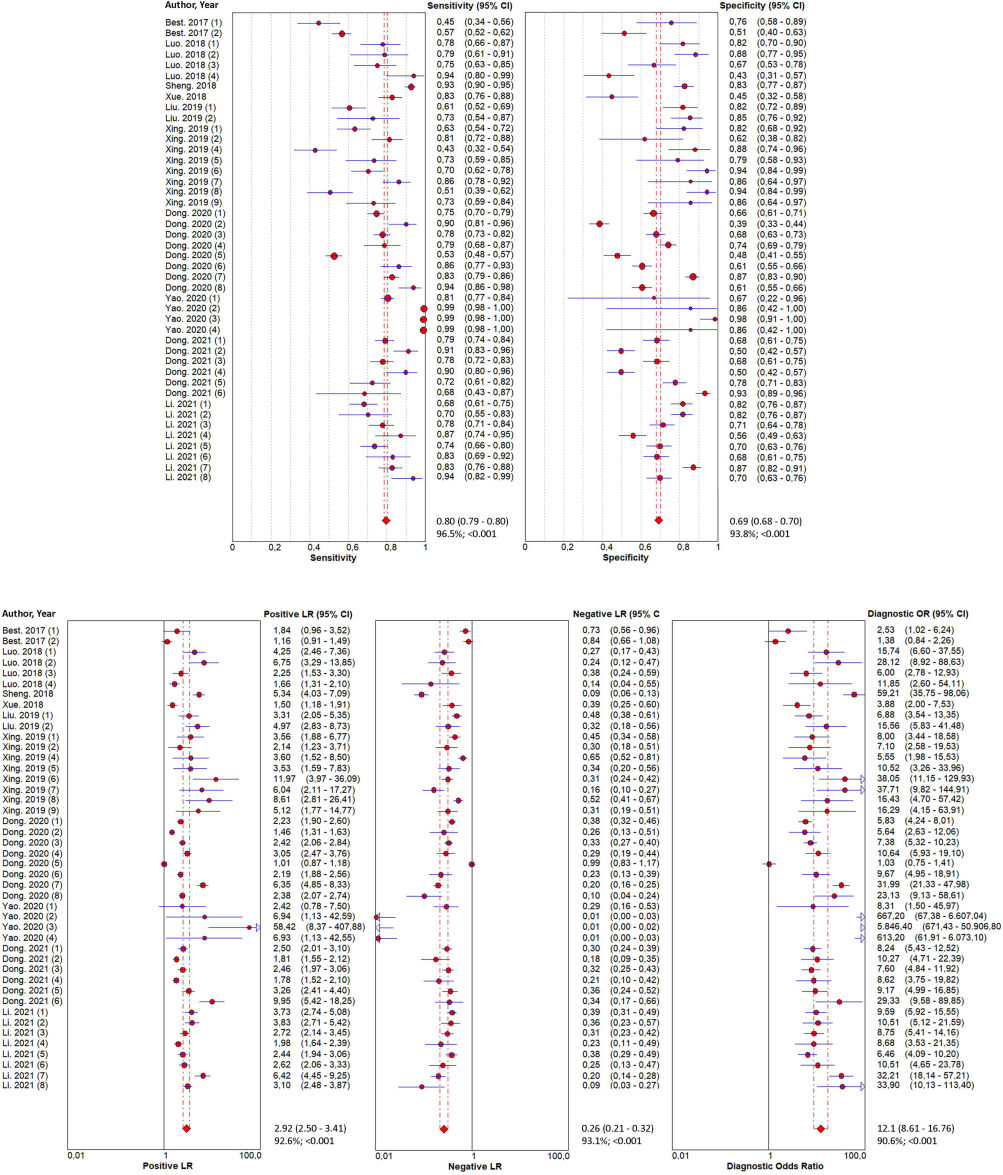
Forest plots of the diagnostic value for tumor-educated platelets in detecting lung cancer. Diagnostic value was analyzed using sensitivity, specificity, positive likelihood ratio, negative likelihood ratio, and diagnostic odds ratio.

**Figure 3:**
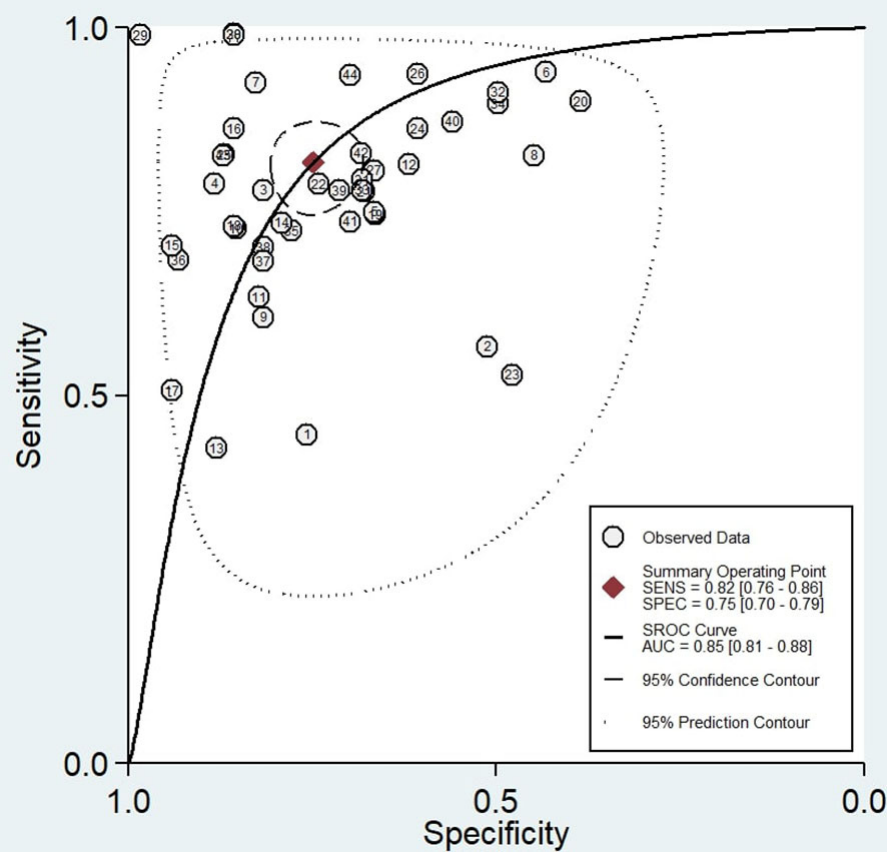
Summary Receiver Operating Characteristic (SROC) curve of tumor-educated platelets in detecting lung cancer.

Forty-four reports from 10 eligible diagnostic studies (21–30) were meta-analyzed and illustrated in **Figure 2**. These plots indicate significant heterogeneity, with I2 values of 96.5%, 93.8%, 92.6%, 93.1%, and 90.6%, respectively, for sensitivity, specificity, PLR, NLR, and DOR; thus, we employed a random effects model in this meta-analysis. The pooled sensitivity, specificity, PLR, NLR, and DOR were 0.80 (95% CI 0.79–0.80), 0.69 (95% CI 0.69–0.70), 2.92 (95% CI 2.50–3.41), 0.26 (95% CI 0.21–0.32), and 12.1 (95% CI 8.61–16.76), respectively. In addition, the AUC of the SROC curve, which indicates diagnostic accuracy, was 0.85 (95% CI: 0.81-0.88), indicating that the diagnostic value of TEPs was moderate (**Figure 3**).

### Heterogeneity and sensitivity analysis

**Figure 4:**
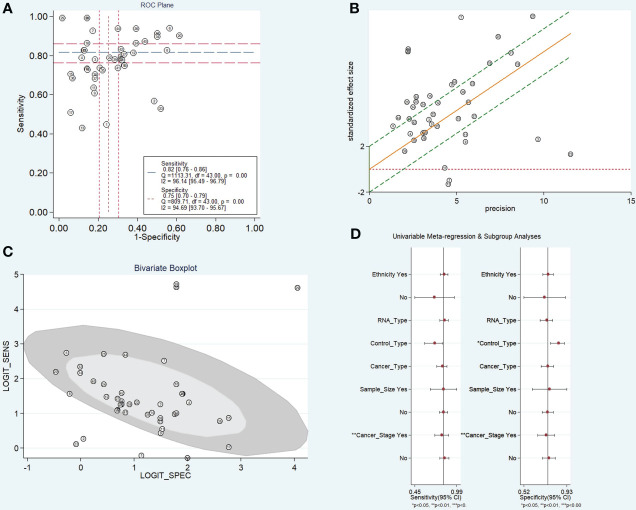
Heterogeneity analysis of diagnostic tests. **(A)** Receiver Operating Characteristic plane. **(B)** Galbraith plot. **(C)** Bivariate boxplot. **(D)** Subgroup and meta-regression analysis for heterogeneity.

As illustrated in **Figure 2**, apparent heterogeneity was found in the pooled sensitivity (I2 = 96.5%, P < 0.001), specificity (I2 = 93.8%, P < 0.001), PLR (I2 = 92.6%, P < 0.001), NLR (I2 = 93.1%, P < 0.001), and DOR (I2 = 90.6%, P < 0.001). To find the potential source of this heterogeneity, we carried out the ROC plane, Galbraith plot, bivariate boxplot, sensitivity analysis, subgroup analysis, and meta-regression analysis. No typical shoulder arm was observed in the ROC plane (**Figure 4A**), indicating that the threshold effect produced no significant heterogeneity. Twenty-two of the 44 reports in the Galbraith plot (**Figure 4B**) and 7 of the 44 reports in the bivariate boxplot (**Figure 4C**) fell outside the 95% CI. These results show that some of these reports may have influenced this review’s heterogeneity. To confirm this result, we conducted a sensitivity analysis to determine the stability of our results using goodness-of-fit, bivariate normality, influence analysis, and outlier detection (**Supplementary Material S7**). The sensitivity analysis results show six reports from three studies (26–28) that mainly affect heterogeneity. After excluding these six reports, the I2 for heterogeneity decreased concerning sensitivity (96.5% to 89.9%) and specificity (93.8% to 91.6%). The overall results showed only minimal changes. To analyze the potential source of heterogeneity, we carried out further subgroup analysis (**Table 2**) and meta-regression analysis (**Figure 4D**). All studies were grouped according to ethnicity (Caucasian and Chinese), RNA type (mRNA, lncRNA, snRNA, circRNA, and snoRNA), control type (HS, BPN, and HS+BPN), cancer type (NSCLC, AD, SCC, and LC), sample size (<100 and ≥100), and cancer stage (early and unspecified). We found that the cancer stage and control type may have accounted for part of the heterogeneity, with p < 0.01 and p < 0.01 for sensitivity and specificity in the cancer stage and p < 0.01 for specificity in the control type. Furthermore, the subgroup analysis based on the control type in the BPN group showed a decrease in I2 to 45.5% and 31.9%, respectively, for sensitivity and specificity.

**Table 2:** Subgroup analysis of the diagnostic efficacy of tumor-educated platelet in lung cancer.

Parameter	No. Report	No. Patient	Sensitivity (95% CI)	Heterogeneity (I^2^; P-value)	Specificity (95% CI)	Heterogeneity (I^2^; P-value)
Ethnicity						
Caucasian	3 (21, 23)	1090	0.721 (0.690-0.751)	98.9%; <0.001	0.750 (0.700-0.795)	92.8%; <0.001
Chinese	41 [22, 24–30]	13151	0.805 (0.796-0.814)	96.0%; <0.001	0.688 (0.677-0.700)	93.9%; <0.001
RNA Type						
mRNA	11 (24–26)	1534	0.695 (0.666-0.723)	87.8%; <0.001	0.805 (0.768-0.839)	83.2%; <0.001
lncRNA	12 (22, 30)	2904	0.778 (0.752-0.803)	66.3%; 0.001	0.728 (0.707-0.748)	89.8%; <0.001
snRNA	8 (27)	4735	0.730 (0.709-0.749)	95.9%; <0.001	0.637 (0.619-0.655)	97.1%; <0.001
circRNA	4 (28)	2227	0.942 (0.932-0.952)	98.7%; <0.001	0.937 (0.858-0.979)	62.6%; 0.046
snoRNA	6 (29)	1882	0.803 (0.773-0.831)	71.9%; 0.003	0.678 (0.650-0.705)	96.3%; <0.001
Control Type						
HS	36 (21–25, 27–30)	13494	0.807 (0.798-0.816)	96.8%; <0.001	0.684 (0.672-0.695)	94.5%; <0.001
BPN	4 (26)	392	0.803 (0.754-0.846)	**45.5%; 0.138**	0.782 (0.680-0.863)	**31.9%; 0.221**
HS+BPN	4 (26)	604	0.590 (0.541- 0.638)	84.2%; <0.001	0.899 (0.847-0.938)	38.8%; 0.179
Cancer Type						
NSCLC	25 (21, 23, 26–29)	9925	0.803 (0.793-0.813)	97.9%; <0.001	0.665 (0.650-0.680)	94.4%; <0.001
AD	4 (22, 29)	906	0.794 (0.752-0.832)	**52.3%; 0.098**	0.627 (0.582-0.669)	88.6%; <0.001
SCC	4 (22, 29)	659	0.776 (0.704-0.838)	65.2%; 0.035	0.807 (0.770-0.841)	95.6%; <0.001
LC	11 (24, 25, 30)	3000	0.761 (0.736-0.785)	78.1%; <0.001	0.732 (0.712-0.753)	90.3%; <0.001
Sample Size						
<100	4 (22, 26)	332	0.784 (0.714-0.844)	60.7%; 0.054	0.703 (0.627-0.772)	91.1%; <0.001
≥100	40 (21–30)	14158	0.796 (0.787-0.805)	96.8%; <0.001	0.691 (0.680-0.702)	94.1%; <0.001
Cancer Stage						
Early	17 (21, 25–27, 29, 30)	4135	0.755 (0.728-0.780)	90.9%; <0.001	0.645 (0.628-0.662)	95.4%; <0.001
Unspecified	27 (21–30)	10355	0.802 (0.793-0.812)	97.5%; <0.001	0.731 (0.716-0.746)	91.1%; <0.001

AD, adenocarcinoma; BPN, benign pulmonary nodule; CI, confidence interval; circRNA, circular ribonucleic acid; HS, healthy subject; LC, lung cancer; lncRNA, long non-coding ribonucleic acid; mRNA, messenger ribonucleic acid; NSCLC, non-small cell lung cancer; RNA, ribonucleic acid; SCC, squamous cell carcinoma; snoRNA, small nucleolar ribonucleic acid; snRNA, small nuclear ribonucleic acid.

### Clinical value of TEPs for LC and publication bias

**Figure 5:**
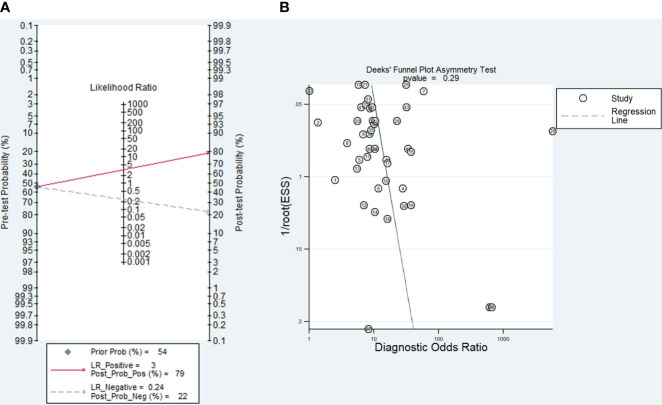
**(A)** Fagan’s nomogram exploring the tumor-educated platelets’ clinical utility in lung cancer with the corresponding **(B)** Deek’s funnel plot.

According to Fagan’s nomogram (**Figure 5A**), the positive post-test probability of diagnosing LC would rise to 79%, while the negative post-test probability would drop to 22%, with a pre-test probability of 54%. The pre-test probability is calculated based on the prevalence of the event group in the total included study (**Table 1**). The P value of Deeks funnel plot asymmetry test (**Figure 5B**) was 0.29, showing no publication bias across the studies.

## Discussion

TEPs broadened the spectrum of liquid biopsy applications and may enable blood-based cancer diagnostics, especially in LC (31). Although several other biomarkers, such as carcinoembryonic antigen (CEA) and Cytokeratin-19 fragment (CYFRA21-1), are commonly used to diagnose LC (32–36), they have low diagnostic effectiveness for the initial stages of lung cancer (34, 37–44). TEPs offer the advantages of faster results, a less invasive nature, and a more convenient technique for diagnosing LC (22). The advantages of TEPs, compared to other liquid biopsy techniques, are related to their abundance, high stability in blood, and ease of isolation (45). Despite TEPs being the relatively more straightforward liquid biopsy methods, the applications of TEPs profiles are still in early development, which may require a lengthy process from biomarker discovery, and design verification, to approval (46, 47). This shortcoming and the high cost of low-input deep sequencing (48), hinder the clinical application of TEPs for detecting LC (46). Nonetheless, TEPs have a potential role as a biomarker for diagnosing LC, which is comprehensively discussed in this review.

As seen in **Table 1**, most of the studies included have substantially diverse results, which lead to inconclusive diagnostic value and clinical utility of TEPs. Each study analyzed in this review used a distinct control group and examined varying stages of lung cancer, all while employing different types of RNA to diagnose the disease. Most of the research included in this review enrolled HS as the control group (21–25, 27–30), while one study enrolled patients with BPN (26). Several studies have also used populations of different stages of cancer, starting from the early stage to the late stage, with non-uniform proportions (21–30). It is also evident that all studies employ diverse RNA types, including mRNA, rRNA, miRNA, snRNA, snoRNA, asRNA, tRNA, circRNA, and lncRNA (28, 48). In patients with cancerous tumors, a particular type of RNA may interact differently than other RNA (29). The results of RNA expression measurement from the same RNA family can show different results (25, 29, 30). Furthermore, it is believed that multiple pathways are involved in the formation of TEPs, including direct communication between tumor cells and platelets, the transmission of information from tumor cells to platelets through extracellular vesicles, and the influence of tumor cells on megakaryocytes (29). Due to the inconclusive results of the various included studies, which were summarized qualitatively, the assessment of the diagnostic value and clinical utility of TEPs was continued with meta-analysis, a more objective assessment.

In the present meta-analysis, screening was performed on 44 reports from 10 eligible studies. The pooled sensitivity, specificity, PLR, NLR, DOR, and SROC AUC results indicated that TEPs have a moderate diagnostic accuracy for LC. The sensitivity analysis confirmed the consistency of the findings, and the Deeks funnel plot asymmetry test demonstrated no apparent publication bias. The Fagan diagram also illustrates its clinical application advantages, primarily attributable to its moderately high positive and negative predictive values.

However, the pooled results must be interpreted cautiously because this review has apparent heterogeneity. Heterogeneity analysis was carried out to find the cause of the emergence of heterogeneity. The ROC plane was utilized to determine whether heterogeneity resulted from the threshold effect. The results demonstrated an atypical shoulder arm, indicating that TEPs have no threshold effect. Six reports from three studies contributed to the significant heterogeneity in TEPs analyses, as indicated by the Galbraith plot, bivariate boxplot, and sensitivity analysis. After excluding these reports, the I2 for heterogeneity decreased. In addition, meta-regression analysis of TEPs revealed that cancer stage and control type may have contributed to the high degree of heterogeneity. Other factors, disparities in measuring equipment and the use of various types of RNA across studies, may also contribute to this heterogeneity.

Heterogeneity due to cancer stages may be associated with the cancer stage and could be linked to the characteristics of platelet RNA in the advancement of tumors (49). For instance, numerous mRNA molecules exhibited different expressions in the platelets of individuals with localized and metastatic cancer (49). Additionally, gene expression was upregulated from the early to the later stage of cancer (49). We also found that the control type attributed to our study’s overall heterogeneity. Some studies used patients with BPN as the control arm, which could lead to heterogeneity because BPN is a constellation of diseases that may result from numerous inflammatory conditions (e.g., tuberculosis, pneumonia, pulmonary abscess), each having different baseline conditions (26).

Our subgroup analysis categorized reports based on different types of RNA used. Each type of RNA has its specific function in gene expression that occurs during tumor cell cycle (31, 50, 51). Also, previous studies reported that tumor-specific signaling in patients with cancer led to distinct RNA processing compared to healthy donors, resulting in numerous variations of genetics between each type of RNA. This finding may result in substantial heterogeneity in our study results (31, 50, 51). We also highlighted the potential effect of different measuring equipment across studies. The results between different measuring equipment may not be comparable due to different data measurements.

According to Fagan’s nomogram, our findings further demonstrate the solid clinical value of TEPs, as evidenced by a 25% increase and a 32% decrease in post-test probability values. This finding suggests that TEPs possess a robust diagnostic capability for LC detection. Given its less invasive nature (51), another potential of TEPs in monitoring and prognosticating NSCLC warrants further investigation. TEPs offer superior clinical applications compared to other liquid biopsy approaches, as platelet isolation is an economical, straightforward process routinely performed for many years (52).

Nonetheless, several limitations of this meta-analysis should be highlighted, both from the evidence included and the review process conducted. First, there was substantial heterogeneity among the included studies and in the subgroup analysis. Second, most of the studies included in this meta-analysis analyzed data from Asian cohorts, and there is a dearth of information regarding TEPs of other ethnicities. Lastly, TEPs are a recently discovered tumor biomarker, and only a limited number of studies (10 studies) can be included in the meta-analysis. This results in incorporating its reports (44 reports in total), which reduces the robustness of certain aggregated analysis results.

This review has implications for clinical practice, future research, and policy. This review demonstrates that TEPs can be a reliable diagnostic instrument for LC in clinical practice. To anticipate the rapid development of science, policies regarding the withdrawal of TEPs in patients with LC can be initiated as early as feasible. In addition, to resolve heterogeneity in this research, future studies on TEPs in LC must have a narrower focus based on factors that influence heterogeneity, e.g., cancer stage, cancer control, methods used, and RNA type. Future studies must conduct TEPs research on specific LC populations, focusing, for instance, on diagnosing early-stage LC compared to HS patients using standardized methodologies for a particular RNA type. It is anticipated that future studies will be able to provide a more uniform picture of the population for implementing TEPs. In addition, in order to accommodate the external validity and generalizability of TEP in lung cancer, further studies are needed to analyze individual datasets, especially with diagnostic study design.

## Conclusion

TEPs could be a moderately effective candidate biomarker for LC diagnosis. This review establishes an essential standard for using TEPs as biomarkers in the early detection of LC. Due to potential limitations, additional research is necessary to corroborate the diagnostic value and clinical utility of TEPs in LC.

## Data availability statement

The original contributions presented in the study are included in the article/**Supplementary material**. Further inquiries can be directed to the corresponding author.

## Author contributions

Conceptualization, EW, GG, and MA. Methodology, EW, DN, MR. Software, GG and MA. Validation, HE, NK, and MM. Formal analysis, EW, DN, and MR. Investigation, GG and MA. Resources, EW. Data curation, EW. Writing—original draft preparation, EW and NK. Writing—review and editing, all authors. Visualization, EW. Supervision, EB and ES. Project administration, EW. Funding acquisition, EB and ES. All authors contributed to the article and approved the submitted version.
