# Supplementary material for: Clinical utility and diagnostic value of tumor-educated platelets in lung cancer: a systematic review and meta-analysis
Source: Front Oncol. 2023 Jul 26;13:1201713. doi: 10.3389/fonc.2023.1201713 (PMC10410284; doi:10.3389/fonc.2023.1201713)
Supplement: Supplementary file 1 [file DataSheet_1.docx]

**S1.** Search strategy

| Database | Keywords |
| --- | --- |
| PubMed | ("lung neoplasms"[MeSH Terms] OR ("lung"[All Fields] AND "neoplasms"[All Fields]) OR "lung neoplasms"[All Fields] OR ("lung"[All Fields] AND "cancer"[All Fields]) OR "lung cancer"[All Fields]) AND ("tumor educated platelet"[All Fields] OR "liquid biopsy"[All Fields]) AND ("sensitivity and specificity"[MeSH Terms] OR ("sensitivity"[All Fields] AND "specificity"[All Fields]) OR "sensitivity and specificity"[All Fields] OR "specificity"[All Fields] OR "specific"[All Fields] OR "specifically"[All Fields] OR "specification"[All Fields] OR "specifications"[All Fields] OR "specificities"[All Fields] OR "specifics"[All Fields] OR "specifities"[All Fields] OR "specifity"[All Fields] OR ("sensitive"[All Fields] OR "sensitively"[All Fields] OR "sensitives"[All Fields] OR "sensitivities"[All Fields] OR "sensitivity and specificity"[MeSH Terms] OR ("sensitivity"[All Fields] AND "specificity"[All Fields]) OR "sensitivity and specificity"[All Fields] OR "sensitivity"[All Fields]) OR ("area under curve"[MeSH Terms] OR ("area"[All Fields] AND "under"[All Fields] AND "curve"[All Fields]) OR "area under curve"[All Fields] OR ("area"[All Fields] AND "under"[All Fields] AND "curve"[All Fields]) OR "area under the curve"[All Fields]) OR (("diagnosis"[MeSH Terms] OR "diagnosis"[All Fields] OR "diagnostic"[All Fields] OR "diagnostical"[All Fields] OR "diagnostically"[All Fields] OR "diagnostics"[All Fields]) AND ("accuracies"[All Fields] OR "accuracy"[All Fields]))) |
| Cochrane CENTRAL | #1 MeSH descriptor: [Lung Neoplasms] explode all trees  #2 (tumor educated platelet OR tumor educate platelet OR tumor educated platelets OR TEP OR platelet RNA OR biological marker OR biomarker OR platelet biomarker OR platelet biological marker OR RNA biomarker OR RNA biological marker):ti,ab,kw (Word variations have been searched)  #3 MeSH descriptor: [Liquid Biopsy] explode all trees  #4 #2 OR #3  #5 (sensitivity OR specificity OR AUC OR area under the curve OR diagnostic accuracy):ti,ab,kw  #6 MeSH descriptor: [Sensitivity and Specificity] explode all trees  #7 #5 OR #6  #7 #1 AND #4 AND #7 |
| Ovid EMBASE | 1. ‘lung cancer’/exp 2. ‘lung cancer’ 3. ‘lung carcinoma’/exp 4. ‘lung carcinoma’ 5. 1 or 2 or 3 or 4 6. ‘tumor educated platelet’/exp 7. ‘tumor educated platelet’ 8. ‘tumor-educated platelet’ 9. ‘thrombocyte’/exp 10. ‘platelet’ 11. 6 or 7 or 8 or 9 or 10 12. ‘sensitivity’/exp 13. ‘sensitivity’ 14. ‘specificity’/exp 15. ‘specificity’ 16. ‘area under the curve’/exp 17. ‘area under the curve’ 18. 12 or 13 or 14 or 15 or 16 or 17 19. 5 and 11 and 18 |
| EBSCO CINAHL | 1. AB lung cancer OR AB lung neoplasm* OR AB lung tumor OR AB lung adenocarcinoma 2. AB Tumor educated platelet OR AB platelet RNA OR AB liquid biopsy 3. AB Specificity OR AB Sensitivity OR AB Area under the curve OR AB Diagnostic Accuracy 4. S3 AND S4 AND S5 |
| Ovid MEDLINE | 1. exp Lung Cancer/ 2. Lung Cancer.mp. 3. 1 or 2 4. Tumor educated platelet.mp. 5. platelet rna.mp. 6. liquid biopsy.mp. 7. 4 or 5 or 6 8. sensitivity.mp. 9. specificity.mp. 10. area under the curve.mp. 11. diagnostic accuracy.mp. 12. 8 or 9 or 10 or 11 13. 3 and 7 and 12 |
| Scopus | ( TITLE-ABS-KEY ( lung AND cancer ) OR TITLE-ABS-KEY ( lung AND neoplasm* ) ) AND ( TITLE-ABS-KEY ( tumor AND educated AND platelet ) OR TITLE-ABS-KEY ( platelet AND rna ) OR TITLE-ABS-KEY ( liquid AND biopsy ) ) AND ( TITLE-ABS-KEY ( sensitivity ) OR TITLE-ABS-KEY ( specificity ) OR TITLE-ABS-KEY ( area AND under AND the AND curve ) OR TITLE-ABS-KEY ( area AND under AND curve ) OR TITLE-ABS-KEY ( diagnostic ) ) AND ( LIMIT-TO ( DOCTYPE , "ar" ) ) |
| ProQuest | (noft(Lung cancer) OR noft(Lung neoplasma) OR noft(Lung tumor) OR noft(Lung adenocarcinoma)) AND (noft(Tumor educated platelet) OR noft(Platelet RNA) OR noft(Liquid biopsy)) AND (noft(Specificity) OR noft(Sensitivity) OR noft(Diagnostic Accuracy)) |
| MedRxiv | ("tumor” AND “educated” AND “platelet”) AND ("lung cancer" OR "lung carcinoma" OR "lung neoplasm") |
| BioRxiv | ("tumor” AND “educated” AND “platelet”) AND ("lung cancer" OR "lung carcinoma" OR "lung neoplasm") |
| SSRN | Platelet RNA |
| Clinicaltrials.gov | Condition or disease: Lung Cancer OR lung neoplasm OR lung carcinoma  Other terms: tumor educated platelet OR platelet RNA |
| CNKI | “Lung Cancer” AND (“Platelet RNA” OR “Tumor educated platelet”) |

CENTRAL: Cochrane Controlled Register of Trials; CINAHL: Cumulative Index to Nursing and Allied Health Literature; CNKI: China National Knowledge Infrastructure; SSRN: Social Science Research Network.
