# Supplementary material for: Clinical utility and diagnostic value of tumor-educated platelets in lung cancer: a systematic review and meta-analysis
Source: Front Oncol. 2023 Jul 26;13:1201713. doi: 10.3389/fonc.2023.1201713 (PMC10410284; doi:10.3389/fonc.2023.1201713)
Supplement: Supplementary file 2 [file DataSheet_2.docx]

**S2.** PICO framework

| Item | Definition |
| --- | --- |
| Population | Lung cancer patients, not limited to age, not limited to comorbidities |
| Intervention | Tumor-educated platelet |
| Comparator | Control |
| Outcome | Specificity, sensitivity, area under the curve, diagnostic accuracy |
