# Supplementary material for: Clinical utility and diagnostic value of tumor-educated platelets in lung cancer: a systematic review and meta-analysis
Source: Front Oncol. 2023 Jul 26;13:1201713. doi: 10.3389/fonc.2023.1201713 (PMC10410284; doi:10.3389/fonc.2023.1201713)
Supplement: Supplementary file 3 [file DataSheet_3.docx]

| Author, year | Country | Ethnicity | Group | Group Type | Total SampleS | Age (mean [SD] or median [range]) | Gender | | Cancer Stage |
| --- | --- | --- | --- | --- | --- | --- | --- | --- | --- |
|  |  |  |  |  |  |  | **Male** | **Female** |  |
| Best, 2017 | Netherlands | Caucasian | Event | NSCLC | 402 | 55 (17- 88) | 376 (49.33%) | 403 (51.7%) | NR |
|  |  |  | Control | HS | 377 |  |  |  | N/A |
| Luo, 2018 | China | Chinese | Event | AD | 68 | <60 years old (48)  ≥60 years old (53) | 74 (73.3%) | 27 (26.7%) | Stage I-II: 25 (24.8%)  Stage III-IV: 76 (75.2%) |
|  |  |  | Event | SCC | 33 |  |  |  |  |
|  |  |  | Control | HS | 60 | NR | NR | NR | N/A |
| Sheng, 2018 | Netherlands | Caucasian | Event | NSCLC | 402 | NR | NR | NR | NR |
|  |  |  | Control | HS | 231 | NR | NR | NR | N/A |
| Xue, 2018 | China | Chinese | Event | LC | 156 | 62.5 (30-89) | 111 (71.2%) | 45 (28.8%) | Stage I: 11 (7.1%)  Stage II: 3 (1.9%)  Stage III: 40 (25.6%)  Stage IV: 65 (41.7%)  Limited Stage: 15 (9.6%)  Extensive Stage: 22 (14.1%) |
|  |  |  | Control | HS | 58 | 58.5 (40-73) | 27 (46.6%) | 31 (53.4%) | N/A |
| Liu, 2019 | China | Chinese | Event | LC | 127 | 62 (36-71) | NR | NR | N/A |
|  |  |  | Control | HS | 89 | NR | NR | NR | N/A |
| Xing, 2019 | China | Chinese | Event | NSCLC | 91 | 60 (53-65) | 54 | 37 | Stage I: 41 (45%)  Stage II-IV: 50 (55%) |
|  |  |  | Control | BPN | 53 | NR | NR | NR | N/A |
|  |  |  | Control | HS | 32 | NR | NR | NR | N/A |
| Dong, 2020 | China | Chinese | Event | NSCLC | 405 | ≥62 years old (199)  <62 years old (183) | NR | NR | Stage 0: 12 (2.9%) Stage I: 68 (16.8%) Stage II: 26 (6.3%) Stage III: 92 (22.8%) Stage IV: 198 (49.0%) Unknown: 9 (2.3%) |
|  |  |  | Control | HS | 204 |  | NR | NR | N/A |
| Yao, 2020 | China | Chinese | Event | NSCLC | 1258 | 63.4 (9.1) | 848 (67.4%) | 410 (32.6%) | Stage I: 728 (57.9%) Stage II: 187 (14.9%) Stage III: 176 (13.9%) Stage IV: 167 (13.3%) |
|  |  |  | Control | HS | 14 | 60.5 (9.1) | 7 (50.0%) | 7 (50.0%) | N/A |
| Dong, 2021 | China | Chinese | Event | NSCLC | 290 | 62 (29-89) | 184 (63.4%) | 106 (36.6%) | Stage 0: 9 (3.1%)  Stage I: 58 (20%)  Stage II: 24 (8.3%)  Stage III: 60 (20.7%)  Stage IV: 135 (46.6%)  Unknown: 4 (1.4%) |
|  |  |  | Control | HS | 189 | 41 (29-87) | 72 (38.1%) | 117 (61.9%) | N/A |
| Li, 2021 | China | Chinese | Event | LC | 329 | >62 years old (311)  ≤62 years old (352) | 436 (69.3%) | 227 (36.1%) | Stage 0: 9 (2.87%) Stage I: 57 (17.36%) Stage II: 20 (6.03%) Stage III: 78 (23.68%) Stage IV: 153 (46.46%) Unknown: 12 (3.62%) |
|  |  |  | Control | HS | 300 |  |  |  | N/A |

AD: Adenocarcinoma; BPN: Benign Pulmonary Nodules; HS: healthy subject; LC: Lung CancerN/A: not applicable; NR: not reported; NSCLC: Non-Small Cell Lung Cancer; SCC: Squamous Cell Carcinoma; SD; standard deviation.
