# Supplementary material for: Clinical utility and diagnostic value of tumor-educated platelets in lung cancer: a systematic review and meta-analysis
Source: Front Oncol. 2023 Jul 26;13:1201713. doi: 10.3389/fonc.2023.1201713 (PMC10410284; doi:10.3389/fonc.2023.1201713)
Supplement: Supplementary file 4 [file DataSheet_4.docx]

| Author, year | Platelet Isolation Method | RNA Quality Assessment Method | RNA Detection Method | RNA Extraction Method | Reverse Transcriptation Method | PCR Method |
| --- | --- | --- | --- | --- | --- | --- |
| Best, 2017 | Centrifugation at 120 g for 20 min, then 360 g for 20 min | Crystal-Violet staining | QiAMP DNA Blood Mini Kit (Qiagen, Hilden, Germany) | mirVana total RNA isolation kit (ThermoFisher, Waltham, US) | Truseq DNA Sample Prep Kit (Illumina, San Diego, US) | SMARTer Ultra Low RNA Kit Illmina Sequencing (Illumina, San Diego, US) |
| Luo, 2018 | Centrifugation at 120 g for 20 min, then 360 g for 20 min | NR | NR | Total RNA Isolation Kit (BioTeke, Beijing, China) | PrimeScript RT reagent Kit (Takara Bio, Kusatsu, Japan) | BioRad CFX96 (Bio-Rad Laboratories, Hercules, US) |
| Sheng, 2018 | Centrifugation at 120 g for 20 min, then 360 g for 20 min | Crystal-Violet staining | QiAMP DNA Blood Mini Kit (Qiagen, Hilden, Germany) | mirVana total RNA isolation kit (ThermoFisher, Waltham, US) | Truseq DNA Sample Prep Kit (Illumina, San Diego, US) | SMARTer Ultra Low RNA Kit Illmina Sequencing (Illumina, San Diego, US) |
| Xue, 2018 | Natural sedimentation for 2 hours, then centrifugation (not specified) | Electrophoresis | Spectrophotometer ASP-3700 | NR | NR | LightCycler 480 Instrument II (Roche, Vaud, Switzerland) |
| Liu, 2019 | Centrifugation at 120 g for 10 min, then 360 g for 20 min | Wright-Giemsa staining | RNeasy Mini kit (Qiagen, Hilden, Germany) | Trizol reagent | PrimeScript RT reagent Kit (Takara Bio, Kusatsu, Japan) | LightCycler 480 Instrument II (Roche, Vaud, Switzerland) |
| Xing, 2019 | Centrifugation at 100 g for 20 min, then 10,000 g for 20 min | Sysmex XN2000 haematology analyser (Sysmex, Asia Green, Singapore) | MGIEasy mRNA library kit (MGI, Shenzhen, China) | Trizol reagent | SuperScript II reverse transcriptase (ThermoFisher, Waltham, US) | LightCycler 480 Instrument II (Roche, Vaud, Switzerland) |
| Dong, 2020 | Centrifugation at 120 g for 10 min, then 360 g for 20 min | NR | NR | Trizol reagent | PrimeScript RT reagent Kit (Takara Bio, Kusatsu, Japan) | LightCycler 480 Instrument II (Roche, Vaud, Switzerland) |
| Yao, 2020 | Centrifugation at 300 g for 30 min, then 800 g for 20 min | Crystal-Violet staining | CircRNA microarray (Arraystar, Rockville, US) | RNeasy Mini Kit (Qiagen, Hilden, Germany) | PrimeScript RT reagent Kit (Takara Bio, Kusatsu, Japan) | Applied Biosystems 7300 Squence Detection System (Applied Biosystems, Foster, US) |
| Dong, 2021 | Centrifugation at 120 g for 10 min, then 360 g for 20 min | Liu staining | NR | Trizol reagent | PrimeScript RT reagent Kit (Takara Bio, Kusatsu, Japan) | LightCycler 480 Instrument II (Roche, Vaud, Switzerland) |
| Li, 2021 | Centrifugations at 120 g for 10 mins, then 360g for 20 mins | Wright-Giemsa staining | LncMRNA microarray (Arraystar, Rockville, US) | Trizol reagent | PrimeScript RT reagent Kit (Takara Bio, Kusatsu, Japan) | LightCycler 480 Instrument II (Roche, Vaud, Switzerland) |

NR: not reported; RNA: ribonucleic acid; RT: reverse transcriptase.
