# Supplementary material for: Clinical utility and diagnostic value of tumor-educated platelets in lung cancer: a systematic review and meta-analysis
Source: Front Oncol. 2023 Jul 26;13:1201713. doi: 10.3389/fonc.2023.1201713 (PMC10410284; doi:10.3389/fonc.2023.1201713)
Supplement: Supplementary file 5 [file DataSheet_5.docx]

| Author, year | Gene Symbol | RNA Type | RNA Forward Primer | RNA Reverse Primer | RNA Control | Control Forward Primer | Control Reverse Primer |
| --- | --- | --- | --- | --- | --- | --- | --- |
| Best, 2017 | thromboSeq | vary | N/A | N/A | NR | NR | NR |
| Luo, 2018 | MAGI2-AS3 (100505881, Gene) | lncRNA | 5'-GAGCAGAAATAGCGGGACCT-3' | 5'-TCTCTTGGATGCAAACGGCA-3' | GAPDH | 5'-GGTCTCCTCTGACTTCAACA-3' | 5'-GTGAGGGTCTCTCTCTTCCT-3' |
|  | ZFAS1 (441951, Gene) | lncRNA | 5'-ACGTGCAGACATCTACAACCT-3' | 5'-TACTTCCAACACCCGCAT-3' | GAPDH | 5'-GGTCTCCTCTGACTTCAACA-3' | 5'-GTGAGGGTCTCTCTCTTCCT-3' |
| Sheng, 2018 | 48-genes biomarker RNA panel | vary | NR | NR | NR | NR | NR |
| Xue, 2018 | ACIN1 (22985, Gene) | mRNA | 5'-AGGTTAGGCAAGGAGGTGGT -3' | 5'-TGTTCCCAAG AGAAGGCTGT-3' | ACTB | 5'-TTAGTTGCGTTACACCCTTTC-3' | 5'-GCTGTCACCTTCACCGTTC-3' |
| Liu, 2019 | MAX (NG_029830.1, GenBank) | mRNA | 5'-CCGAGGTTTCAATCTGCGG-3' | 5'-GAGGTCGTCAATATCTTGCTGG-3' | ACTB | 5'-CTGGAAGGTGGACAGCGAGG-3' | 5'-TGACGTGGACATCCGCAAAG-3' |
|  | MTURN (222166, Gene) | mRNA | 5'-CGCAGGATGGATTTCTACGC-3' | 5'-GCCCCAGTAAAGGTCTGAAAG-3' | ACTB | 5'-CTGGAAGGTGGACAGCGAGG-3' | 5'-TGACGTGGACATCCGCAAAG-3' |
|  | HLA‐B (3106, Gene) | mRNA | 5'-CAGTTCGTGAGGTTCGACAG-3' | 5'-CAGCCGTACATGCTCTGGA-3' | ACTB | 5'-CTGGAAGGTGGACAGCGAGG-3' | 5'-TGACGTGGACATCCGCAAAG-3' |
| Xing, 2019 | ITGA2B (NM_000419.4, GenBank) | mRNA | 5’-CTTTGACCTCCGTGATGAGACC-3’ | 5’-CAGTCTTTTCTAGGACGTTCCAGTG-3’ | RPL32 | 5’-TCAAGGAGCTGGAAGTGCTG- 3’ | 5’-CATTGGGGTTGGTGACTC TG-3’ |
|  | SELP (GenBank NM_003005.3) | mRNA | 5’-TGGCAAGTGGAATGATGAGC-3’ | 5’-GCAGGTGTAGTTCCCGATGG-3’ | RPL32 | 5’-TCAAGGAGCTGGAAGTGCTG- 3’ | 5’-CATTGGGGTTGGTGACTC TG-3’ |
| Dong, 2020 | RNU1 (26871, Gene) | snRNA | 5'-CAGGGGAGATACCATGATCACGAAG-3' | 5'-CGCAGTCCCCCACTACCACAAAT-3' | 18S | 5'-GGCCCTGTAATTGGAATGAGTC-3' | 5'-CCAAGATCCAACTACGAGCTT-3' |
|  | RNU2 (6066, Gene) | snRNA | 5'-CCTTTTGGCTAAGATCAAGTGTAGTATCTGTT-3' | 5'-AGCAAGCTCCTATTCCATCTCCCTG-3' | 18S | 5'-GGCCCTGTAATTGGAATGAGTC-3' | 5'-CCAAGATCCAACTACGAGCTT-3' |
|  | RNU5 (URS0000635FD4_9606, RNAcentral) | snRNA | 5'-TACTCTGGTTTCTCTTCAGATCGCATAA-3' | 5'-CTCAAAAAATTGGGTTAAGACTCAGA-3' | 18S | 5'-GGCCCTGTAATTGGAATGAGTC-3' | 5'-CCAAGATCCAACTACGAGCTT-3' |
| Yao, 2020 | CD274 (29126, Gene) | circRNA | NR | NR | NR | NR | NR |
|  | ITGA2B (NM_000419.4, GenBank) | circRNA | NR | NR | NR | NR | NR |
|  | TIMP1 (7076, Gene) | circRNA | NR | NR | NR | NR | NR |
|  | FLNA (2316, Gene) | circRNA | NR | NR | NR | NR | NR |
| Dong, 2021 | SNORD55 (26811, Gene) | snoRNA | 5′-GACAACTCGGTAATGCTGCATACTC-3′ | 5′-GCTCTCCAAGGTTGGCTTCCC-3′ | U6 | 5′-TGGAACGCTTCACGAATTTGCG-3′ | 5′-GGAACGATACAGAGAAGATTAGC-3′ |
| Li, 2021 | linc-GTF2H2-1 (TCONS_L2_00022946, UCSC) | lncRNA | 5'-TTGTGGACAGGATGGTGATTGCTC-3' | 5'-GTCTGCTGCGTAGGTGGAGTTG-3' | ACTB | 5'-CTGGAAGGTGGACAGCGAGG-3' | 5'-TGACGTGGACATCCGCAAAG-3' |
|  | RP3-466P17.2 (ENST00000603042, GENCODE) | lncRNA | 5'-ACACAACATAGGCAGCACAGT-3' | 5'-AGTCATTTGGCGATGCAGAGA-3' | ACTB | 5'-CTGGAAGGTGGACAGCGAGG-3' | 5'-TGACGTGGACATCCGCAAAG-3' |
|  | lnc-ST8SIA4-12 (LNC-ST8SIA4-12:1, LNCipedia) | lncRNA | 5'-CTTCGGCTCATTTCTTGGCG-3' | 5'-TGAGAAGGCAGTTGAAGCGT-3' | ACTB | 5'-CTGGAAGGTGGACAGCGAGG-3' | 5'-TGACGTGGACATCCGCAAAG-3' |

circRNA: circular ribonucleic acid; lncRNA; long non-coding ribonucleic acid; mRNA: messenger ribonucleic acid; NR: not reported; RNA: ribonucleic acid; snoRNA: small nucleolar ribonucleic acid; snRNA: small nuclear ribonucleic acid.
