# Supplementary material for: Clinical utility and diagnostic value of tumor-educated platelets in lung cancer: a systematic review and meta-analysis
Source: Front Oncol. 2023 Jul 26;13:1201713. doi: 10.3389/fonc.2023.1201713 (PMC10410284; doi:10.3389/fonc.2023.1201713)
Supplement: Supplementary file 6 [file DataSheet_6.docx]

**Risk of bias and applicability concerns graph: review authors' judgements about each domain presented as percentages across included studies**

**
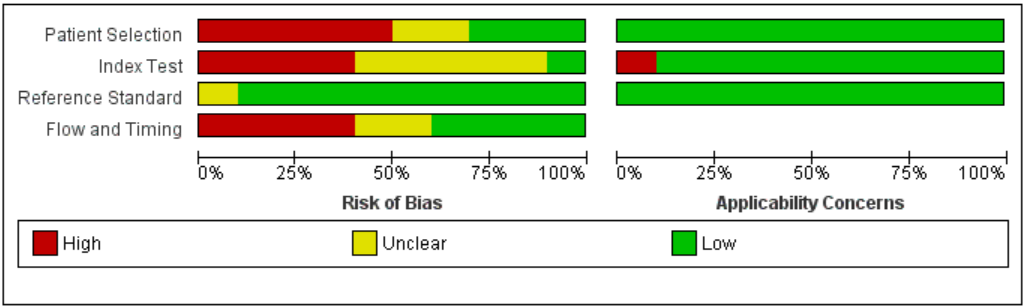
**

**Risk of bias and applicability concerns summary: review authors' judgements about each domain for each included study**

**
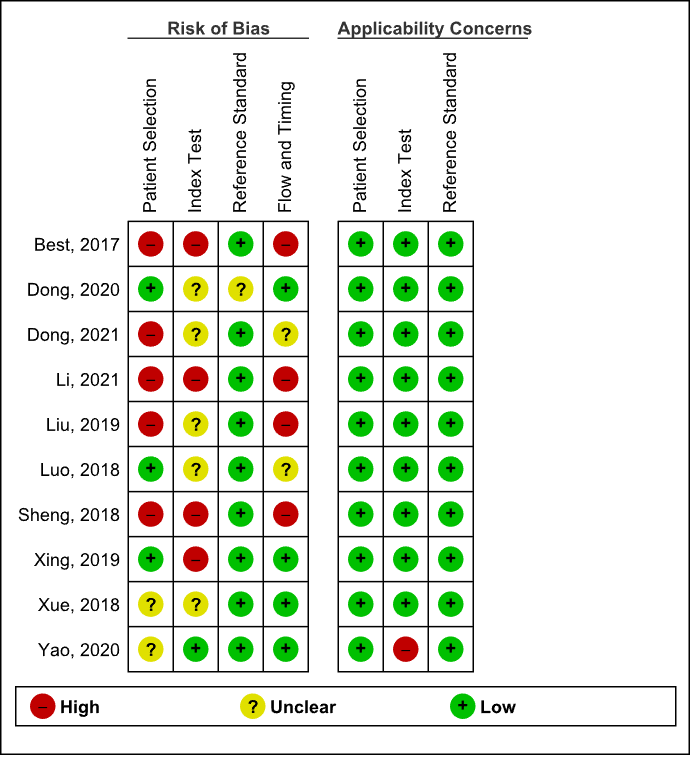
**

**RISK OF BIAS**

**Risk of Bias**

| Best, 2017 | | |
| --- | --- | --- |
| **Patient selection: Could the selection of patients have introduced bias?**  **RISK: HIGH** | | |
| Question | Judgment | Reasoning |
| Was a consecutive or random sample of patients enrolled? | Yes | Enrollment of patients was done consecutively based on study inclusion criteria. |
| Was a case-control design avoided? | No | The study was conducted by obtaining patients with non-small cell lung carcinoma (NSCLC) as cases and individuals with no known cancer as control. |
| Did the study avoid inappropriate exclusions? | Yes | The study did not have major exclusion criteria. Instead, extrinsic factors that may influence results were categorized in study subcohorts. |
| **Index test: Could the conduct or interpretation of the index test have introduced bias?**  **RISK: HIGH** | | |
| Question | Judgment | Reasoning |
| Were the index test results interpreted without knowledge of the results of the reference standard? | No | The author had already known the results of the reference standard prior to interpretation of index test results. |
| If a threshold was used, was it pre-specified? | No | The study did not use a threshold to define negative/positive results. |
| **Reference standard: Could the reference standard, its conduct, or its interpretation have introduced bias?**  **RISK: LOW** | | |
| Question | Judgment | Reasoning |
| Is the reference standard likely to correctly classify the target condition? | Yes | Reference standard used in this study is pathological confirmation of lung cancer, which has been known as the gold standard of cancer diagnosis. |
| Were the reference standard results interpreted without knowledge of the results of the index test? | Yes | Reference standard results were already available from the beginning of the study. |
| **Flow and timing: Could the patient flow have introduced bias?**  **RISK: HIGH** | | |
| Question | Judgment | Reasoning |
| Was there an appropriate interval between index test and reference standard? | Unclear | The study did not mention a specific interval between interpretation of index test and reference standard. |
| Did all patients receive the same reference standard? | No | Reference standard was only performed in those with clinical suspicion of lung cancer. |
| Were all patients included in the analysis? | Unclear | The author did not report the exact research flow, thus information regarding drop-outs were not available. |

**Applicability**

| Best, 2017 | | |
| --- | --- | --- |
| Question | Judgment | Reasoning |
| **Are there concerns that the included patients and setting do not match the review question?** | Low | This study included patients with non-small-cell lung cancer (NSCLC), which aligns with our review question. |
| **Are there concerns that the index test, its conduct, or interpretation differ from the review question?** | Low | This study used a particle-swarm optimization (PSO)-enhanced algorithm to conduct the index test, which differs from most methods used in other studies. However, the principle mechanism of the index test is relatively similar to our review question, which focuses on evaluation of TEP as a biomarker for lung cancer. |
| **Are there concerns that the target condition as defined by the reference standard does not match the question?** | Low | Pathological examination is the main diagnostic tool widely used to confirm the diagnosis of lung cancer as our target condition. |

**Risk of Bias**

| Luo, 2018 | | |
| --- | --- | --- |
| **Patient selection: Could the selection of patients have introduced bias?**  **RISK: LOW** | | |
| Question | Judgment | Reasoning |
| Was a consecutive or random sample of patients enrolled? | Yes | The authors did not explicitly mention the sampling method, but the sample appears to be consecutive based on the article's context. |
| Was a case-control design avoided? | No | The study included patients with pathologically confirmed NSCLC (cases) and healthy individuals (controls). The authors compared the expression levels of lncRNAs and EGFRvIII in TEPs between the two groups in a case-control design. |
| Did the study avoid inappropriate exclusions? | Unclear | Inclusion and exclusion criteria of the subjects were not stated. |
| **Index test: Could the conduct or interpretation of the index test have introduced bias?**  **RISK: UNCLEAR** | | |
| Question | Judgment | Reasoning |
| Were the index test results interpreted without knowledge of the results of the reference standard? | Unclear | The authors do not provide specific details on whether they blinded the index test results to the reference standard results during their analysis. |
| If a threshold was used, was it pre-specified? | No | The study aimed to evaluate the diagnostic value of lncRNAs and EGFRvIII in TEPs for NSCLC patients compared to healthy controls. The authors reported the expression levels of these biomarkers and their diagnostic performance based on sensitivity, specificity, and area under the curve (AUC) in a receiver operating characteristic (ROC) curve analysis. However, they do not mention a pre-specified threshold for determining positive or negative test results. |
| **Reference standard: Could the reference standard, its conduct, or its interpretation have introduced bias?**  **RISK: LOW** | | |
| Question | Judgment | Reasoning |
| Is the reference standard likely to correctly classify the target condition? | Yes | Given that the reference standard used in this study is pathological confirmation, it is likely to correctly classify the target condition (NSCLC) in the patients included in the study. |
| Were the reference standard results interpreted without knowledge of the results of the index test? | Unclear | The authors do not explicitly state whether the reference standard results were interpreted without knowledge of the results of the index test. |
| **Flow and timing: Could the patient flow have introduced bias?**  **RISK: UNCLEAR** | | |
| Question | Judgment | Reasoning |
| Was there an appropriate interval between index test and reference standard? | Unclear | The authors do not provide specific details about the timing of the index tests and reference standard. As a result, it is difficult to determine if there was an appropriate interval between the two tests in this study. |
| Did all patients receive the same reference standard? | No | In the context of this study, the focus is on comparing the expression levels of lncRNAs and EGFRvIII in tumor-educated platelets (TEPs) between NSCLC patients and healthy controls. The use of a separate reference standard for the healthy control group is not applicable, as they are not expected to have the target condition (NSCLC). |
| Were all patients included in the analysis? | Unclear | The authors do not explicitly discuss whether there were any missing data or excluded participants during the analysis. |

**Applicability**

| Luo, 2018 | | |
| --- | --- | --- |
| Question | Judgment | Reasoning |
| Are there concerns that the included patients and setting do not match the review question? | Low | While the authors do not provide extensive details about the patient demographics or the clinical setting, the study's focus on comparing lncRNAs and EGFRvIII expression levels in TEPs between NSCLC patients and healthy controls aligns with the research question. |
| Are there concerns that the index test, its conduct, or interpretation differ from the review question? | Low | Based on the information provided in the article, there do not appear to be major concerns that the index test, its conduct, or interpretation differ from the review question. The authors focus on measuring lncRNAs and EGFRvIII levels in TEPs, which is consistent with the research question. However, some aspects of the study, such as the threshold used for determining positive or negative test results, are not explicitly mentioned. Despite these limitations, the overall conduct and interpretation of the index test seem to align with the research question. |
| Are there concerns that the target condition as defined by the reference standard does not match the question? | Low | The target condition is non-small cell lung cancer (NSCLC), and the reference standard used for its diagnosis is pathological confirmation. The reference standard, pathological confirmation, is a well-established and reliable method for diagnosing cancer, including NSCLC. The use of pathological confirmation as the reference standard is appropriate for the study's objective. |

**Risk of Bias**

| Sheng, 2018 | | |
| --- | --- | --- |
| **Patient selection: Could the selection of patients have introduced bias?**  **RISK: HIGH** | | |
| Question | Judgment | Reasoning |
| Was a consecutive or random sample of patients enrolled? | No | Enrollment of patients was done from previously recorded samples. |
| Was a case-control design avoided? | No | The study used previously recorded blood gene-expression profiles of patients with non-small cell lung carcinoma (NSCLC) as cases and individuals with no known cancer as control. |
| Did the study avoid inappropriate exclusions? | No | The study excluded samples with chronic pancreatitis, epilepsy, multiple sclerosis, insignificant atherosclerosis, pulmonary hypertension, stable angina pectoris, and unstable angina pectoris from the original study without further explanation. |
| **Index test: Could the conduct or interpretation of the index test have introduced bias?**  **RISK: HIGH** | | |
| Question | Judgment | Reasoning |
| Were the index test results interpreted without knowledge of the results of the reference standard? | No | The author should have already known the results of the index test results from the previous study, prior to interpretation of index test results. |
| If a threshold was used, was it pre-specified? | No | The study did not use a pre-specified threshold to define negative/positive results. |
| **Reference standard: Could the reference standard, its conduct, or its interpretation have introduced bias?**  **RISK: LOW** | | |
| Question | Judgment | Reasoning |
| Is the reference standard likely to correctly classify the target condition? | Yes | Reference standard used in the previous study whose result is reused in the study is pathological confirmation of lung cancer, which has been known as the gold standard of cancer diagnosis. |
| Were the reference standard results interpreted without knowledge of the results of the index test? | Yes | Reference standard results were already available from the beginning of the study. |
| **Flow and timing: Could the patient flow have introduced bias?**  **RISK: HIGH** | | |
| Question | Judgment | Reasoning |
| Was there an appropriate interval between index test and reference standard? | Unclear | The study did not mention a specific interval between interpretation of index test and reference standard as it used a previously recorded study with known results of reference standard. |
| Did all patients receive the same reference standard? | No | Reference standard was only performed in those with clinical suspicion of lung cancer. |
| Were all patients included in the analysis? | No | The author of the reused data did not report the exact research flow, and this study excluded even more samples. |

**Applicability**

| Sheng, 2018 | | |
| --- | --- | --- |
| Question | Judgment | Reasoning |
| **Are there concerns that the included patients and setting do not match the review question?** | Low | This study included patients with non-small-cell lung cancer (NSCLC), which aligns with our review question. |
| **Are there concerns that the index test, its conduct, or interpretation differ from the review question?** | High | This study used a 48-gene biomarker panel derived using minimal redundancy, maximal relevance (MRMR) and incremental feature selection (IFS), and then leave-one-out cross-validation (LOOCV) was used to predict the performance of the panel. Despite its similarity of principle mechanism to our review question, the usage of prediction to derive actual results differs from our target question. |
| **Are there concerns that the target condition as defined by the reference standard does not match the question?** | Low | Pathological examination, which is used in the study that is reused in this study, is the main diagnostic tool widely used to confirm the diagnosis of lung cancer as our target condition. |

**Risk of Bias**

| Xue, 2018 | | |
| --- | --- | --- |
| **Patient selection: Could the selection of patients have introduced bias?**  **RISK: UNCLEAR** | | |
| Question | Judgment | Reasoning |
| Was a consecutive or random sample of patients enrolled? | Unclear | The process of patient selection is inadequately explained, and the paper does not provide any details regarding the inclusion or exclusion criteria. |
| Was a case-control design avoided? | Unclear |  |
| Did the study avoid inappropriate exclusions? | Unclear |  |
| **Index test: Could the conduct or interpretation of the index test have introduced bias?**  **RISK: UNCLEAR** | | |
| Question | Judgment | Reasoning |
| Were the index test results interpreted without knowledge of the results of the reference standard? | Unclear | It is not explicitly stated in the provided information whether the index test results were interpreted without knowledge of the results of the reference standard. |
| If a threshold was used, was it pre-specified? | No | The pre-specification of the threshold for index test is not apparent as it is only mentioned in the "Outcome" section. |
| **Reference standard: Could the reference standard, its conduct, or its interpretation have introduced bias?**  **RISK: LOW** | | |
| Question | Judgment | Reasoning |
| Is the reference standard likely to correctly classify the target condition? | Yes | Yes, histopathological is sufficient to classify the target condition (lung cancer). |
| Were the reference standard results interpreted without knowledge of the results of the index test? | Unclear | It is not explicitly stated in the provided information whether the index test results were interpreted without knowledge of the results of the reference standard. |
| **Flow and timing: Could the patient flow have introduced bias?**  **RISK: LOW** | | |
| Question | Judgment | Reasoning |
| Was there an appropriate interval between index test and reference standard? | Unclear | The paper does not specify the time interval between the index test and the reference standard. |
| Did all patients receive the same reference standard? | Yes | As shown in Fig. 2 |
| Were all patients included in the analysis? | Unclear | While the tables present data for 156 patients and 58 controls, the discussion section indicates that the expression level of ACIN1 mRNA was analyzed in platelets from 146 lung cancer patients and 58 healthy controls in this study. There is a probability where this happens as a typographical error. |

**Applicability**

| Xue, 2018 | | |
| --- | --- | --- |
| Question | Judgment | Reasoning |
| Are there concerns that the included patients and setting do not match the review question? | Concern: Low | While the authors do not provide extensive details about the patient demographics or the clinical setting, the study's focus on comparing ACIN1 mRNA expression levels in TEPs between lung cancer patients and healthy controls aligns with the research question. |
| Are there concerns that the index test, its conduct, or interpretation differ from the review question? | Concern: Low | The index test used in the study (ACIN1 mRNA expression levels in platelets) appears to be relevant and aligned with the research question. The study aims to investigate the potential of ACIN1 mRNA in platelets as a biomarker for lung cancer, and the index test used is the detection of ACIN1 mRNA expression levels in platelets. |
| Are there concerns that the target condition as defined by the reference standard does not match the question? | Concern: Low | There do not appear to be any concerns that the target condition as defined by the reference standard does not match the review question. The study aims to explore the potential of ACIN1 mRNA in platelets as a biomarker for lung cancer, and the reference standard used is the pathological examination of lung tissue, which is a well-established method for diagnosing lung cancer. |

**Risk of Bias**

| Liu, 2019 | | |
| --- | --- | --- |
| **Patient selection: Could the selection of patients have introduced bias?**  **RISK: HIGH** | | |
| Question | Judgment | Reasoning |
| Was a consecutive or random sample of patients enrolled? | Unclear | The authors did not explicitly mention the sampling method, but the sample appears to be consecutive based on the article's context |
| Was a case-control design avoided? | No | This study included already diagnose lung cancer patients admitted to Shandong Cancer Hospital and Institute and healthy individuals as controls. |
| Did the study avoid inappropriate exclusions? | No | The number of samples in cohort study regarding UQCRH, MAX, MTURN, HLA-B expression is different with total sample |
| **Index test: Could the conduct or interpretation of the index test have introduced bias?**  **RISK: UNCLEAR** | | |
| Question | Judgment | Reasoning |
| Were the index test results interpreted without knowledge of the results of the reference standard? | No | The author had already known the results of the reference standard prior to interpretation of index test results. |
| If a threshold was used, was it pre-specified? | Unclear | It is not stated whether threshold was used or not |
| **Reference standard: Could the reference standard, its conduct, or its interpretation have introduced bias?**  **RISK: LOW** | | |
| Question | Judgment | Reasoning |
| Is the reference standard likely to correctly classify the target condition? | Yes | Reference standard used in this study were clinical, pathological and radiological examination which has been used as the gold standard to diagnoses lung cancer. |
| Were the reference standard results interpreted without knowledge of the results of the index test? | Yes | Reference standard results were already available from the beginning of the study |
| **Flow and timing: Could the patient flow have introduced bias?**  **RISK: HIGH** | | |
| Question | Judgment | Reasoning |
| Was there an appropriate interval between index test and reference standard? | No | The study did not mention a specific interval between interpretation of index test and reference standard. |
| Did all patients receive the same reference standard? | No | This study evaluated the diagnostic capability of platelet m-RNA in lung cancer compared to healthy donors, in which the reference standard test was not applicable to the latter. |
| Were all patients included in the analysis? | No | The number of samples in cohort study regarding UQCRH, MAX, MTURN, HLA-B expression is different with total sample |

**Applicability**

| Liu, 2019 | | |
| --- | --- | --- |
| Question | Judgment | Reasoning |
| **Are there concerns that the included patients and setting do not match the review question?** | Low | This study included patients with lung cancer which aligns with our review question. |
| **Are there concerns that the index test, its conduct, or interpretation differ from the review question?** | Low | The study used LightCycler 480 qPCR system to quantify the expression levels of TEP RNAs as a potential biomarker for lung cancer. The index test, its conduct, and interpretation are similar to our review question. |
| **Are there concerns that the target condition as defined by the reference standard does not match the question?** | Low | Pathological and radiological examination are the diagnostic tool widely used to confirm the diagnosis of lung cancer as our target condition. |

**Risk of Bias**

| Xing, 2019 | | |
| --- | --- | --- |
| **Patient selection: Could the selection of patients have introduced bias?**  **RISK: LOW** | | |
| Question | Judgment | Reasoning |
| Was a consecutive or random sample of patients enrolled? | Yes | Enrollment of patients was done consecutively based on study inclusion criteria. |
| Was a case-control design avoided? | No | The study included patients with pathologically confirmed NSCLC as cases and patients with benign pulmonary nodules and healthy individuals as controls. |
| Did the study avoid inappropriate exclusions? | Yes | The study excluded 25 patients with secondary lung cancer from other primary regions and 6 patients with history of other solid tumors. The exclusion criteria seem appropriate and relevant to the research question. |
| **Index test: Could the conduct or interpretation of the index test have introduced bias?**  **RISK: HIGH** | | |
| Question | Judgment | Reasoning |
| Were the index test results interpreted without knowledge of the results of the reference standard? | No | The author had already known the results of the reference standard prior to interpretation of index test results. |
| If a threshold was used, was it pre-specified? | No | It is not stated whether the cutoff used was pre-specified or not. |
| **Reference standard: Could the reference standard, its conduct, or its interpretation have introduced bias?**  **RISK: LOW** | | |
| Question | Judgment | Reasoning |
| Is the reference standard likely to correctly classify the target condition? | Yes | Reference standard used in this study is pathological examination, which has been used as the gold standard of lung cancer diagnosis. |
| Were the reference standard results interpreted without knowledge of the results of the index test? | No | Reference standard results were already available from the beginning of the study. |
| **Flow and timing: Could the patient flow have introduced bias?**  **RISK: LOW** | | |
| Question | Judgment | Reasoning |
| Was there an appropriate interval between index test and reference standard? | Unclear | The study did not mention a specific interval between interpretation of index test and reference standard. |
| Did all patients receive the same reference standard? | No | The use of histopathological examination as a reference standard for the healthy control group is not applicable. Reference standard was only performed in patients with NSCLC. |
| Were all patients included in the analysis? | Yes | Yes, the research flow shows that all patients were included in the analysis. |

**Applicability**

| Xing, 2019 | | |
| --- | --- | --- |
| Question | Judgment | Reasoning |
| **Are there concerns that the included patients and setting do not match the review question?** | Low | The study included patients with NSCLC, which is similar to our review question. |
| **Are there concerns that the index test, its conduct, or interpretation differ from the review question?** | Low | The study used qRT-PCR to quantify the expression levels of TEP RNAs as a potential biomarker for lung cancer. The index test, its conduct, and interpretation are similar to our review question. |
| **Are there concerns that the target condition as defined by the reference standard does not match the question?** | Low | The reference standard used in the study was histopathological examination, which is a well-established method to diagnose lung cancer. |

**Risk of Bias**

| Dong, 2020 | | |
| --- | --- | --- |
| **Patient selection: Could the selection of patients have introduced bias?**  **RISK: LOW** | | |
| Question | Judgment | Reasoning |
| Was a consecutive or random sample of patients enrolled? | Unclear | It is not explicitly stated whether a consecutive or random sample of patients was enrolled in the study. However, the authors do mention that patients with lung cancer were enrolled from the Department of Oncology at the Affiliated Hospital of Guizhou Medical University. The sample size of 100 patients with lung cancer and 50 healthy controls suggests that the study was likely a convenience sample, which may limit the generalizability of the findings. |
| Was a case-control design avoided? | Yes | The study design and methodology described in the paper suggest that it is likely that a case-control design was avoided. |
| Did the study avoid inappropriate exclusions? | Yes | The study included 382 lung cancer patients without any anticancer treatment and 23 patients with initial treatment, and excluded healthy volunteers who were free from any malignant tumor after examination. The exclusion criteria seem appropriate and relevant to the research question. |
| **Index test: Could the conduct or interpretation of the index test have introduced bias?**  **RISK: UNCLEAR** | | |
| Question | Judgment | Reasoning |
| Were the index test results interpreted without knowledge of the results of the reference standard? | Unclear | The information provided in the results section does not indicate whether the index test results were interpreted without knowledge of the results of the reference standard. |
| If a threshold was used, was it pre-specified? | Unclear | It is not explicitly stated whether it was pre-specified or not. |
| **Reference standard: Could the reference standard, its conduct, or its interpretation have introduced bias?**  **RISK: UNCLEAR** | | |
| Question | Judgment | Reasoning |
| Is the reference standard likely to correctly classify the target condition? | Yes | Histopathological is sufficient to classify the target condition (lung cancer). |
| Were the reference standard results interpreted without knowledge of the results of the index test? | Unclear | The information provided in the results section does not indicate whether the reference standard results were interpreted without knowledge of the results of the index test. |
| **Flow and timing: Could the patient flow have introduced bias?**  **RISK: LOW** | | |
| Question | Judgment | Reasoning |
| Was there an appropriate interval between index test and reference standard? | Unclear | The paper does not specify the time interval between the index test and the reference standard. |
| Did all patients receive the same reference standard? | Yes | Cancer diagnosis was determined through a histological examination of tumor specimens of all patients. |
| Were all patients included in the analysis? | Yes | It appears that all patients enrolled in the study were included in the analysis. |

**Applicability**

| Dong, 2020 | | |
| --- | --- | --- |
| Question | Judgment | Reasoning |
| Are there concerns that the included patients and setting do not match the review question? | Concern: Low | The study included patients with confirmed diagnosis of lung cancer and healthy controls, which is consistent with the review question of validating TEP snRNAs as potential biomarkers for lung cancer. The study was also conducted in a clinical setting, which is relevant to the review question. |
| Are there concerns that the index test, its conduct, or interpretation differ from the review question? | Concern: Low | The study used a well-established technique (qRT-PCR) to quantify the expression levels of TEP snRNAs, which is appropriate for the validation of potential biomarkers. |
| Are there concerns that the target condition as defined by the reference standard does not match the question? | Concern: Low | The reference standard used in the study was histopathological analysis, which is a well-established method for the diagnosis of lung cancer. |

**Risk of Bias**

| Yao, 2020 | | |
| --- | --- | --- |
| **Patient selection: Could the selection of patients have introduced bias?**  **RISK: UNCLEAR** | | |
| Question | Judgment | Reasoning |
| Was a consecutive or random sample of patients enrolled? | Unclear | It is not clear whether the participant selection was consecutive or random. The study states that a total of 1473 peripheral blood samples were collected from six groups of participants, including healthy volunteers, patients carrying benign pulmonary nodules, and lung cancer patients, but it does not provide information on how the participants were selected or recruited. |
| Was a case-control design avoided? | Yes | It is a cohort study. |
| Did the study avoid inappropriate exclusions? | Unclear | It is unclear whether the study avoided inappropriate exclusions or not. The study only provided information on the inclusion criteria and did not mention any specific exclusion criteria. |
| **Index test: Could the conduct or interpretation of the index test have introduced bias?**  **RISK: LOW** | | |
| Question | Judgment | Reasoning |
| Were the index test results interpreted without knowledge of the results of the reference standard? | Yes | It is not explicitly stated in the provided information whether the index test results were interpreted without knowledge of the results of the reference standard. However, it is mentioned that the patients' clinical characteristics were blinded during the validation of the four-circRNAs panel (PCs) in predicting the blinded patient samples. This suggests that efforts were made to minimize the risk of bias during the interpretation of the results. |
| If a threshold was used, was it pre-specified? | Unclear | It is not explicitly stated whether it was pre-specified or not. However, it is mentioned that the fold change threshold used to define upregulation of the circRNAs was >2, and the statistical significance threshold was P < 0.01. |
| **Reference standard: Could the reference standard, its conduct, or its interpretation have introduced bias?**  **RISK: LOW** | | |
| Question | Judgment | Reasoning |
| Is the reference standard likely to correctly classify the target condition? | Yes | Yes, the reference standard methods of percutaneous lung puncture, electronic bronchoscope, thoracoscope, or surgery are all considered reliable methods for confirming the histopathological nature of nodules. |
| Were the reference standard results interpreted without knowledge of the results of the index test? | Yes | It is not explicitly stated in the provided information whether the index test results were interpreted without knowledge of the results of the reference standard. However, it is mentioned that the patients' clinical characteristics were blinded during the validation of the four-circRNAs panel (PCs) in predicting the blinded patient samples. This suggests that efforts were made to minimize the risk of bias during the interpretation of the results. |
| **Flow and timing: Could the patient flow have introduced bias?**  **RISK: LOW** | | |
| Question | Judgment | Reasoning |
| Was there an appropriate interval between index test and reference standard? | Unclear | The paper does not specify the time interval between the index test and the reference standard. |
| Did all patients receive the same reference standard? | Yes | All use histopathological examination although the means of obtaining the tissue varies. |
| Were all patients included in the analysis? | Yes | Yes, all patients (1473 blood samples) were included in the analysis. |

**Applicability**

| Yao, 2020 | | |
| --- | --- | --- |
| Question | Judgment | Reasoning |
| Are there concerns that the included patients and setting do not match the review question? | Concern: Low | The study focuses on proving their hypothesis that platelet-circRNA profile that can be used  as a fingerprint for early detection of lung cancer.  The included patients align with the research question. |
| Are there concerns that the index test, its conduct, or interpretation differ from the review question? | Concern: High | The study focused on identifying a panel of circRNAs that were differentially expressed in platelets of lung cancer patients compared to healthy controls. However, the review question asks specifically about the ability of platelet-circRNA profiles to serve as a fingerprint for early detection of lung cancer. It is not clear whether the identified circRNAs could reliably distinguish between early-stage lung cancer and benign lung nodules or other non-cancerous lung conditions. |
| Are there concerns that the target condition as defined by the reference standard does not match the question? | Concern: Low | It appears that the target condition as defined by the reference standard does match the review question, |

**Risk of Bias**

| Dong, 2021 | | |
| --- | --- | --- |
| **Patient selection: Could the selection of patients have introduced bias?**  **RISK: HIGH** | | |
| Question | Judgment | Reasoning |
| Was a consecutive or random sample of patients enrolled? | Unclear | The authors did not explicitly mention the sampling method, but the sample appears to be consecutive based on the article's context |
| Was a case-control design avoided? | No | This study included already diagnose NSCLC patients admitted to Shandong Cancer Hospital and Institute and Shandong Provincial Third Hospital. The control participants were healthy individuals |
| Did the study avoid inappropriate exclusions? | Yes | All participants were included in the analysis |
| **Index test: Could the conduct or interpretation of the index test have introduced bias?**  **RISK: UNCLEAR** | | |
| Question | Judgment | Reasoning |
| Were the index test results interpreted without knowledge of the results of the reference standard? | No | The author had already known the results of the reference standard prior to interpretation of index test results. |
| If a threshold was used, was it pre-specified? | Unclear | It is not stated whether threshold was used or not |
| **Reference standard: Could the reference standard, its conduct, or its interpretation have introduced bias?**  **RISK: LOW** | | |
| Question | Judgment | Reasoning |
| Is the reference standard likely to correctly classify the target condition? | Yes | All patient have been diagnose to a certain stage of NSCLC |
| Were the reference standard results interpreted without knowledge of the results of the index test? | Yes | Reference standard results were already available from the beginning of the study |
| **Flow and timing: Could the patient flow have introduced bias?**  **RISK: UNCLEAR** | | |
| Question | Judgment | Reasoning |
| Was there an appropriate interval between index test and reference standard? | No | The study did not mention a specific interval between interpretation of index test and reference standard. |
| Did all patients receive the same reference standard? | Unclear | All heathy participants have been excluded from any malignant tumor but there were no detail information regarding the examination method |
| Were all patients included in the analysis? | Yes | All NSCLC patient are included in the analysis |

**Applicability**

| Dong, 2021 | | |
| --- | --- | --- |
| Question | Judgment | Reasoning |
| **Are there concerns that the included patients and setting do not match the review question?** | Low | This study included patients with NSCLC which aligns with our review question. |
| **Are there concerns that the index test, its conduct, or interpretation differ from the review question?** | Low | The study used LightCycler 480 qPCR system to quantify the expression levels of snoRNAs as a potential biomarker for lung cancer. The index test, its conduct, and interpretation are similar to our review question. |
| **Are there concerns that the target condition as defined by the reference standard does not match the question?** | Low | All patient have been diagnose to a certain stage of NSCLC using clinicopathological examination |

**Risk of Bias**

| Li, 2021 | | |
| --- | --- | --- |
| **Patient selection: Could the selection of patients have introduced bias?**  **RISK: HIGH** | | |
| Question | Judgment | Reasoning |
| Was a consecutive or random sample of patients enrolled? | Yes | This study used consecutive sampling to obtain both patients with lung cancer and healthy donors. |
| Was a case-control design avoided? | No | This study was conducted in a case-control design, which involved patients with confirmed lung cancer (cases) and healthy donors who did not present with any disease (controls). |
| Did the study avoid inappropriate exclusions? | Unclear | The study did not report any exclusion criteria and whether there were subject exclusion. |
| **Index test: Could the conduct or interpretation of the index test have introduced bias?**  **RISK: HIGH** | | |
| Question | Judgment | Reasoning |
| Were the index test results interpreted without knowledge of the results of the reference standard? | No | The study did not report any blinding approach and the results of the reference standard had been known prior to the index test. |
| If a threshold was used, was it pre-specified? | No | Cut-off was not pre-specified. |
| **Reference standard: Could the reference standard, its conduct, or its interpretation have introduced bias?**  **RISK: LOW** | | |
| Question | Judgment | Reasoning |
| Is the reference standard likely to correctly classify the target condition? | Yes | Lung cancer was diagnosed using a combination of clinical, pathological, and radiological approaches followed by TNM staging with established international standards. |
| Were the reference standard results interpreted without knowledge of the results of the index test? | Yes | Reference standard results were already available from the beginning of the study. |
| **Flow and timing: Could the patient flow have introduced bias?**  **RISK: HIGH** | | |
| Question | Judgment | Reasoning |
| Was there an appropriate interval between index test and reference standard? | No | The interval between index test and reference standard could lead to different results, as lung cancer may progress during the time being (eg., the study included an analysis of TEP accuracy in diagnosing early-stage cancer) |
| Did all patients receive the same reference standard? | No | This study evaluated the diagnostic capability of TEP RNA in lung cancer compared to healthy donors, in which the reference standard test (particularly pathological and radiological examination) was not applicable to the latter. |
| Were all patients included in the analysis? | Yes | All patients were included in the analysis. |

**Applicability**

| Li, 2021 | | |
| --- | --- | --- |
| Question | Judgment | Reasoning |
| **Are there concerns that the included patients and setting do not match the review question?** | Low | This study included patients with lung cancer (irrespective of the cancer type), which aligns with our review question. |
| **Are there concerns that the index test, its conduct, or interpretation differ from the review question?** | Low | The index test, including its conduct and interpretation is similar to our review question. |
| **Are there concerns that the target condition as defined by the reference standard does not match the question?** | Low | Target condition of this study is patients with lung cancer, which were mostly defined with combinations of clinical, radiological, and histopathological examination. |
