# Supplementary figures and images for: Clinical utility and diagnostic value of tumor-educated platelets in lung cancer: a systematic review and meta-analysis
Source: Front Oncol. 2023 Jul 26;13:1201713. doi: 10.3389/fonc.2023.1201713 (PMC10410284; doi:10.3389/fonc.2023.1201713)

**S7.** Sensitivity analysis


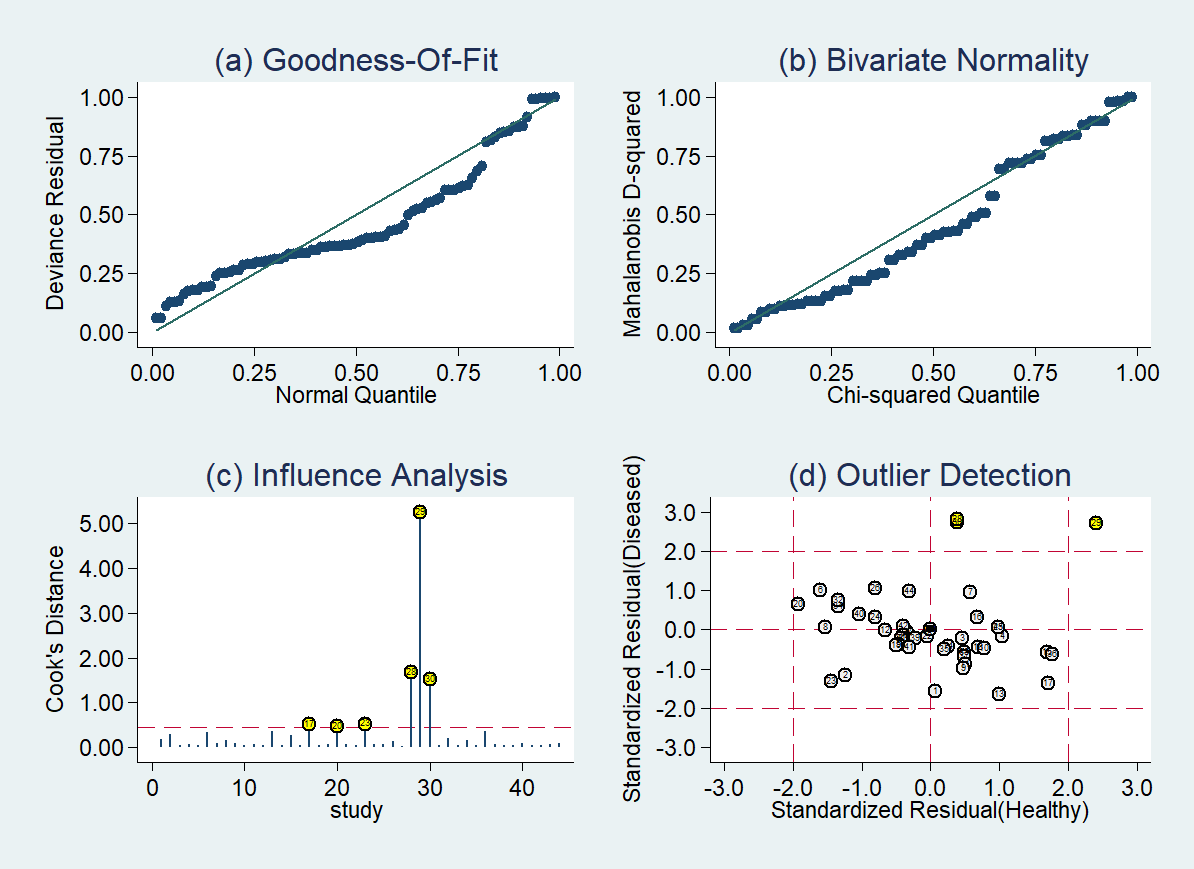

Supplement: Supplementary file 7 [file DataSheet_7.docx]
